# The role of cGAS-STING signaling in rheumatoid arthritis: from pathogenesis to therapeutic targets

**DOI:** 10.3389/fimmu.2024.1466023

**Published:** 2024-09-25

**Authors:** Qiugang Zhu, Huimin Zhou

**Affiliations:** ^1^ Department of Laboratory Medicine, Shangyu People’s Hospital of Shaoxing, Shaoxing University, Shaoxing, China; ^2^ Department of Laboratory Medicine, Wuxi Ninth People’s Hospital Affiliated to Soochow University, Wuxi, China

**Keywords:** rheumatoid arthritis, cGAS, STING, signaling pathway, treatments

## Abstract

Rheumatoid arthritis (RA) is a systemic autoimmune disease primarily characterized by erosive and symmetric polyarthritis. As a pivotal axis in the regulation of type I interferon (IFN-I) and innate immunity, the cyclic GMP-AMP synthase-stimulator of interferon genes (cGAS-STING) signaling pathway has been implicated in the pathogenesis of RA. This pathway mainly functions by regulating cell survival, pyroptosis, migration, and invasion. Therefore, understanding the sources of cell-free DNA and the mechanisms underlying the activation and regulation of cGAS-STING signaling in RA offers a promising avenue for targeted therapies. Early detection and interventions targeting the cGAS-STING signaling are important for reducing the medical burden on individuals and healthcare systems. Herein, we review the existing literature pertaining to the role of cGAS-STING signaling in RA, and discuss current applications and future directions for targeting the cGAS-STING signaling in RA treatments.

## 1 Introduction

Rheumatoid arthritis (RA) is an autoimmune disease caused by the breakdown of immune homeostasis, affecting women more frequently than men. Clinical features of RA primarily include joint swelling, pain, stiffness, weakness, deformity, and fatigue (1). Pathologically, RA is characterized by chronic inflammation of the joint synovium, formation of pannus, and infiltration of lymphocytes, macrophages, and neutrophils (2). Common treatments for RA include non-steroidal anti-inflammatory drugs (NSAIDs), synthetic disease-modifying antirheumatic drugs (DMARDs), biological DMARDs, traditional Chinese medicine, and surgical interventions (3, 4). Representative therapeutic options available for patients include celecoxib, methotrexate (MTX), glucocorticoids, tumor necrosis factor (TNF) inhibitors, IL-6R inhibitors, Janus kinases (JAKs) inhibitors, and anti-B cell antibodies, patients may require multiple drugs with different modes of action to address the heterogeneity of RA (5). Despite these therapies, the clinical symptoms of certain patients remain unrelieved, underscoring the need for a deeper understanding of RA’s pathogenic mechanisms to explore novel treatment options.

Innate immunity plays a critical role in the pathogenesis of RA, including various innate immune cells and components (6). For example, increased expression of toll-like receptors (TLRs, TLR2/3/4/7) has been reported in RA. Ligand-stimulated TLRs activate the intracellular MyD88-dependent and MyD88-independent pathway, resulting in the induction of various pro-inflammatory cytokines in RA (7). An increased interferon gene signature was observed in patients with early RA (eRA), which predicted a poor response to the initial therapies in the first 6 months after diagnosis (8). Also, there was a correlation between baseline interferon gene signature and disease activity score 28 at 6 months. Further exploration demonstrated that interferon-α played an important role in therapeutic resistance by regulating site-specific DNA methylation in B and T cells (9). Thus, dysregulated IFN-I potentially plays a role in the pathogenesis and therapeutic resistance of RA (10).

The cyclic GMP-AMP synthase-stimulator of interferon genes (cGAS-STING) signaling pathway is crucial for cells to recognize and respond to cytosolic double-stranded DNA (dsDNA), serving as a primary driver for the establishment of innate immunity through the induction of IFN-I (11). TNF-α is a pathogenic cytokine in RA, which has been demonstrated to increase DNA damage and nuclear DNA release, accompanied by reduced STING degradation (12). Thus, it is reasonable to speculate that TNF can regulate RA progression through cGAS-STING signaling. Here, we delve into the role of cGAS-STING signaling in RA from the origin of cell-free DNA (cfDNA) to the final effects. Also, the potential therapeutic applications of cGAS-STING signaling in RA treatment will be discussed, aiming to provide new insights for the future research on RA.

## 2 An overview of the cGAS-STING signaling pathway

The cGAS-STING signaling pathway is widely distributed in immune cells, non-immune cells, tumor cells, and other tissue-derived cells (13–15). The primary function of cGAS-STING signaling is to trigger the innate immune response by inducing IFN-I production and subsequent interferon-stimulated gene (ISG) expression (16, 17). This signaling also plays roles in other cellular activities, including autophagy, pyroptosis, metabolism, and cellular senescence (18–21). Moreover, the cGAS-STING signaling can be modulated by cellular molecules, RNA virus-derived components, and post-translational modifications to maintain homeostasis under normal conditions, with its dysregulation potentially contributing to disease development (22–24).

cGAS, acts as a cytosolic DNA sensor, recognizing DNA in the cytoplasm that originates from pathogens, mitochondria, micronuclei, and dead cells (25). The activation of cGAS is triggered by its interactions with dsDNA, which is dependent on the length of DNA (>45 nucleotides) rather than the sequence (26). The availability of longer dsDNA fragments allows for the attainment of a certain signaling threshold (27, 28). Upon cGAS activation, cyclic GMP-AMP (cGAMP) is synthesized from GTP and ATP, which is responsible for eliciting the downstream signaling (29). In addition, the DNA-RNA hybrids can also induce the activation of cGAS (30). It is also noteworthy that cGAS can reside in the nucleus (31). Studies have shown that nuclear cGAS binds to nucleosomes (mainly H2A-H2B), which prevents the cGAS-DNA binding and cGAS dimerization, thereby maintaining cGAS in an inactive conformation and consequently limiting autoreactivity (32–34).

**Figure 1:**
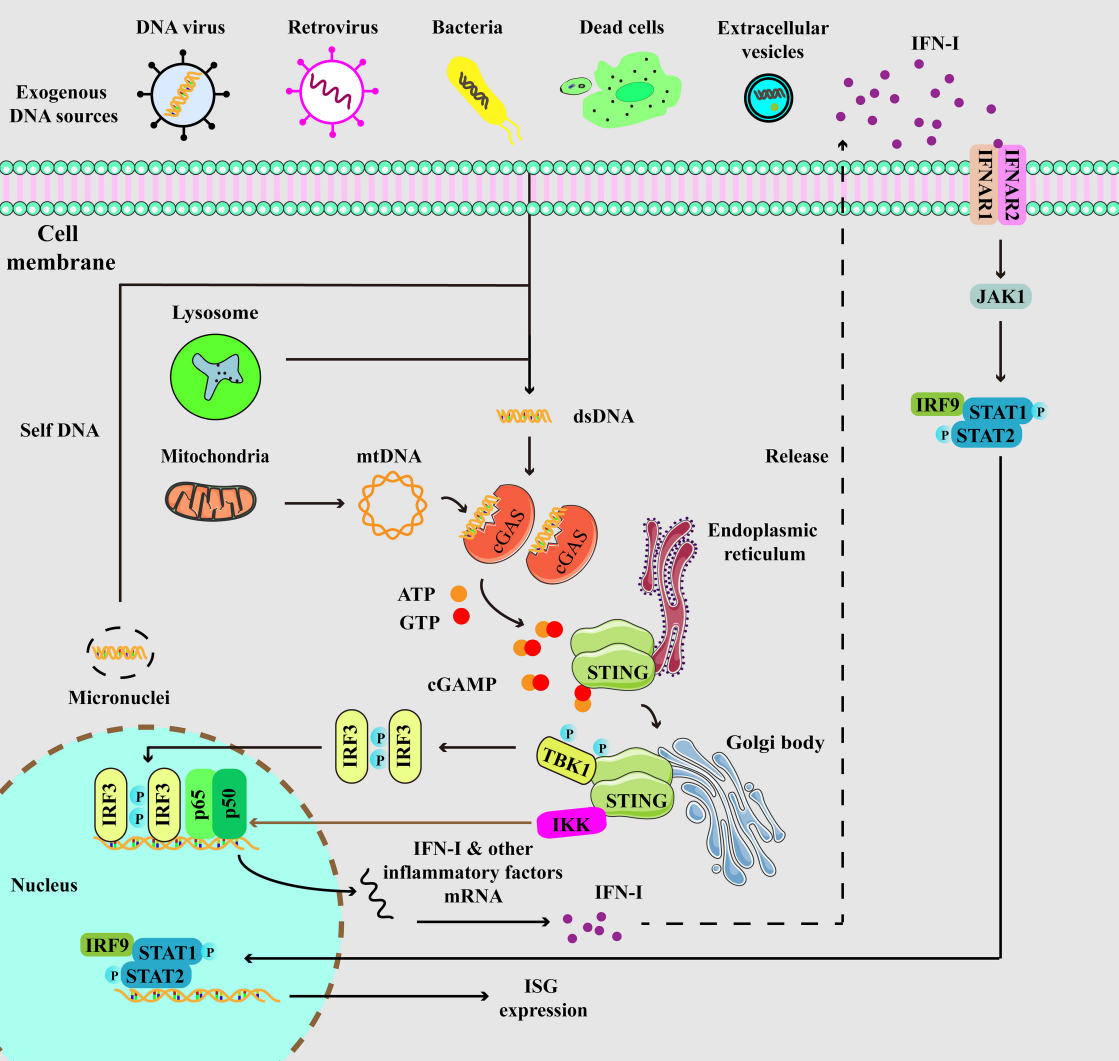
Depiction of cGAS-STING signaling. cGAS, a cytosolic DNA sensor, is able to detect cytoplasmic DNA from exogenous and endogenous sources, including DNA viruses, retrovirus, bacteria, dead cells, extracellular vesicles, micronuclei, and mitochondria. When cGAS binds to double-stranded DNA (dsDNA), it triggers the activation of its own catalytic activity, resulting in the synthesis of 2’,3’-cyclic GMP-AMP (cGAMP) from ATP and GTP. cGAMP binds to STING at the endoplasmic reticulum (ER), then STING undergoes oligomerization and translocates from the ER to Golgi compartments. Then, STING is palmitoylated and serves as a platform for the recruitment of TBK1 and IKK. TBK1 phosphorylates STING, which in turn recruits IRF3 for TBK1-mediated phosphorylation. Phosphorylated IRF3 translocates to the nucleus and turns on the expression of type I interferons. Meanwhile, STING also activates IKK to facilitate NF-κB-mediated transcription of pro-inflammatory cytokines, thereby activating inflammatory responses. Moreover, secreted IFN-I can be recognized by IFNAR, which induces the activation of JAK-STAT signaling, resulting in the expression of IFN-stimulated genes (ISGs).

STING, initially identified before cGAS, is a 379 amino acid protein located on the endoplasmic reticulum (ER) membrane (35, 36). cGAMP binds to STING, resulting in profound conformational changes that trigger STING oligomerization. Subsequently, tetramers of STING translocate to Golgi compartments through the ER-Golgi intermediate compartments (ERGIC). STING then facilitates the recruitment of TANK binding kinase 1 (TBK1), which promotes TBK1 autophosphorylation and STING phosphorylation. This process further triggers the recruitment and phosphorylation of interferon regulatory factor 3 (IRF3). Phosphorylated IRF3 undergoes dimerization and translocates to the nucleus, initiating the expression of IFN-I. Ultimately, IFN-I induces the expression of ISGs through IFNAR. Additionally, STING also induces the activation of IKK, leading to the nuclear entry of nuclear factor-κB (NF-κB) and subsequent the expression of inflammatory factors (**Figure 1**) (16, 17). Interestingly, a previous study revealed that interferon production could be induced by membrane fusion in a STING-dependent but cGAS-independent manner (37).

cGAS-STING signaling activation and IFN-I production are involved in multiple pathological and physiological processes. During viral infections, IFN-I promotes the clearance of the virus, but it may also cause immunosuppression during chronic infections (38). However, excessive expression of IFN-I enhances the autoreactive T cell- and B cell-mediated responses, ultimately resulting in the occurrence of autoimmunity (16, 39). Although other branch activations are also involved in RA, such as the cGAS-PI3K-Akt signaling pathway, this review will primarily focus on the cGAS-STING signaling pathway in the following sections (40, 41).

## 3 cGAS-STING signaling in the progression of RA

### 3.1 Elevated levels of cfDNA, cGAS, and STING in RA

There was a study that systematically reviewed the level and origin of cfDNA in RA (42). As early as 1973, cfDNA was confirmed to be elevated in the serum of RA patients (43). Later, the increased level of cfDNA was also detected in the synovial fluid. Moreover, there was an association between cfDNA levels and disease activity (44, 45). Potential sources of cfDNA include neutrophil extracellular traps/neutrophil ETosis (NETs/NETosis), pyroptosis, and micronuclei (46–48). *In vitro*, RA synovial fluid enhanced the production of NETs by neutrophils, as evidenced by the increased levels of DNA in culture supernatants and extracellular DNA on NETs (48). A significant association between gasdermin E (GSDME) and cfDNA has been observed in RA patients, further experiments have demonstrated that NETs triggered NF-κB/Caspase 3/GSDME-mediated pyroptosis in fibroblast-like synoviocytes (FLSs), indicating that pyroptosis is a source of cfDNA (46). Micronuclei, which also serve as a crucial source of cfDNA, have been found to be elevated in RA patients (47). This increase in micronuclei has been observed in both active and inactive RA patients, accompanied by the reduced levels of superoxide dismutase and glutathione peroxidase (49). Increased micronucleus levels were also detected in collagen-induced arthritis (CIA) mice, a mouse model established by immunization with an emulsion of complete Freund’s adjuvant (CFA) and type II collagen (CII). Notably, rituximab treatment suppressed micronucleus formation, paralleled by a decline in serum 8-hydroxydeoxyguanosine, indicating that enhanced oxidative stress might contribute to DNA damage and micronucleus formation in RA (50). Previous research found that MTX enhanced the generation of micronuclei in rat bone marrow cells (51, 52). In contrast, another study found no difference in micronucleus levels between patients who received MTX treatment and those who did not, suggesting that the generation of micronuclei was associated with RA itself (47). Collectively, both the disease itself and the pharmacological treatments have the potential to induce the formation of micronuclei in RA, thereby providing a basis for the generation of cfDNA. Consequently, the presence of cfDNA may act as the initiator for the activation of cGAS-STING signaling during RA progression.

**Figure 2:**
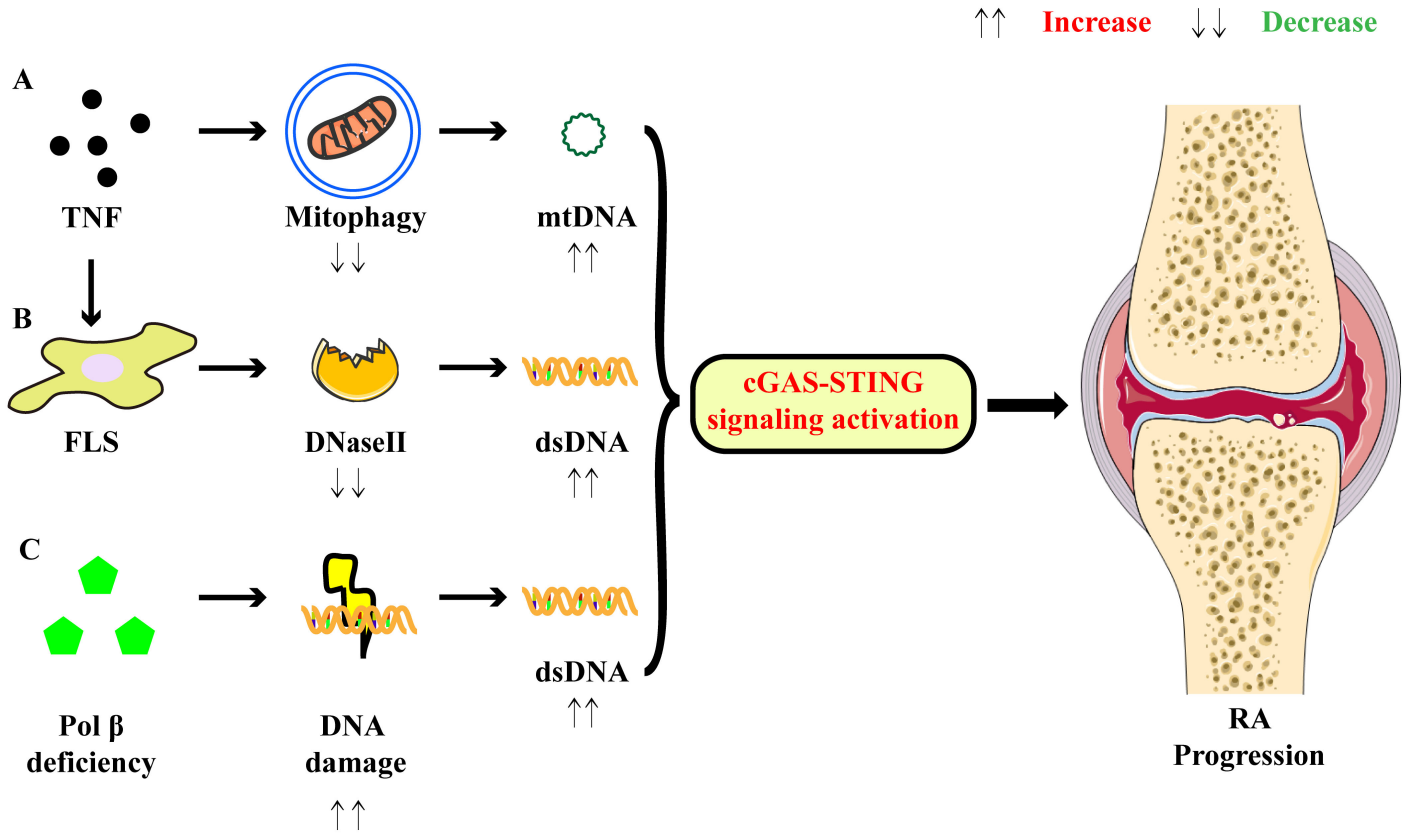
Activation of cGAS-STING signaling promotes the pathogenesis of RA. **(A)** TNF inhibits mitophagy and then results in increased levels of mtDNA. **(B)** TNF suppresses the expression of DNaseII in FLSs, leading to the accumulation of cytosolic dsDNA. **(C)** Pol β deficiency promotes DNA damage and dsDNA leakage, resulting in the increased levels of cytosolic dsDNA. All these contribute to the activation of cGAS-STING signaling and subsequent expression of pro-inflammatory factors, thereby supporting the joint inflammation in RA.

Compared with the osteoarthritis (OA) patient-derived FLSs, the levels of cGAS mRNA and cGAS protein were higher in RA-FLSs. Moreover, overexpression of cGAS in RA-FLSs enhanced both the proliferation of these cells and the expression of pro-inflammatory factors (41). As for STING, RA patients exhibited the highest concentrations of intracellular STING when compared to those with OA, psoriatic arthritis, calcium pyrophosphate crystal-induced arthritis, and OA with calcium pyrophosphate crystals (53). Furthermore, intracellular STING positively correlated with inflammatory parameters, such as white blood cells, polymorphonuclear cells, IL-1β, IL-8, and IL-6 (53). *Sting1^+/−^* mice, which have a reduced expression of phosphorylated TBK1, showed a decreased severity of arthritis and improved histological changes compared to control mice (54). These findings suggest that cfDNA-triggered cGAS-STING signaling plays a significant role in the pathogenesis of RA (**Figure 2**).

### 3.2 TNF triggers the release of mtDNA and the activation of cGAS-STING signaling in RA

TNF, a multifunctional cytokine for homeostasis and disease pathogenesis, is highly expressed in rheumatoid joint tissues (55). TNF is also the first cytokine validated as a therapeutic target for RA, and several types of TNF inhibitors have been applied in the clinical treatment (56). A previous study revealed that TNF could enhance IFN responses by activating cGAS-STING signaling, thereby supporting the joint inflammation (57). Specifically, TNF inhibited PTEN-induced kinase 1 -mediated mitophagy, leading to functional alterations in mitochondria and an increase in cytoplasmic mitochondrial DNA (mtDNA) levels. Consequently, mtDNA bound to cGAS and activated the downstream signaling that mimicked the functions of macrophages from RA patients, contributing to the pathogenesis of RA (57). Additionally, TNF-α stimulation has been demonstrated to increase the expression of cGAS in FLSs, further supporting the involvement of TNF in RA progression through cGAS-STING signaling (41).

Growing evidence has indicated that obesity plays a pivotal role in multiple aspects of RA (58–60). A trend toward increased risk of RA was observed among overweight and obese women, particularly women diagnosed with RA at earlier ages (≤55 years) (59). Moreover, obesity has been revealed to reduce the effectiveness of TNF inhibitors, resulting in lower chances of achieving remission or low disease activity (58, 60). Increased fat mass and obesity-associated protein (FTO) expression has been detected in FLSs from RA patients and synovial cells from adjuvant-induced arthritis (AIA) mice (a mouse model induced by intradermal injection of CFA). The elevated expression of FTO was involved in mtDNA-mediated synovial inflammation (61). In detail, TNF-α-induced mtDNA expression was decreased when FTO was knocked down in RA-FLSs. Furthermore, FTO knockdown suppressed the activation of TNF-α-induced cGAS-STING signaling, accompanied by the decreased expression of inflammatory cytokines such as IL-6 and IL-18, subsequently alleviating AIA (61). Mechanistically, these effects elicited by FTO were dependent on cytidine/uridine monophosphate kinase 2 (CMPK2). The inhibition of CMPK2 expression following FTO reduction led to decreased mtDNA production and cGAS-STING signaling activation, thereby suppressing inflammatory cytokine expression and ameliorating arthritis (61). Therefore, TNF can contribute to the pathogenesis of RA in a cGAS-STING signaling-dependent manner by disrupting mitochondrial homeostasis, and this process can be regulated by FTO.

### 3.3 cGAS-STING signaling participates in the abnormal activation of FLSs

FLSs exhibit abnormal activation and proliferation in the synovium of RA patients, serving as primary effector cells responsible for mediating joint destruction and synovitis (62). *In vitro*, transfection of dsDNA upregulated the expression of IFN-α and IFN-β in RA-FLSs, suggesting that cytosolic dsDNA accumulation enhanced the IFN-I signature. Additionally, stimulation with dsDNA upregulated the production of pro-inflammatory cytokines and matrix metalloproteinase (MMP) 13 in FLSs. TNF-α-induced DNaseII reduction might be responsible for the accumulation of dsDNA in FLSs, as TNF-α stimulation decreased both the mRNA and protein levels of DNaseII (63). Mechanistically, cGAS-STING signaling was implicated in the cytosolic dsDNA-triggered responses in FLSs. Knockdown of cGAS or STING significantly suppressed the dsDNA-induced pro-inflammatory cytokines secretion (63). Additionally, another study demonstrated that dsDNA-triggered cGAS-STING signaling augmented the migratory and invasive capabilities of RA-FLSs, which were suppressed by cGAS or STING short hairpin RNA treatment (64). In this study, scientists found that activation of cGAS-STING signaling increased the levels of mitochondrial reactive oxygen species, which induced the phosphorylation of mammalian sterile 20-like kinase 1 and then activated forkhead box1, subsequently promoting FLS migration and invasion (64). Thus, cGAS-STING signaling appears to be essential for the pathogenic activities of FLSs, and this signaling represents a promising target to prevent the aberrant activation of FLSs for RA treatments.

### 3.4 Other factors regulate cGAS-STING signaling in RA

The cGAS-STING-NF-κB signaling, which represents another arm of the STING signaling network, has been documented to induce macrophage pyroptosis, a process that holds significant importance in RA (65). In this study, they found that DNA polymerase β (Pol β) regulated RA pathogenesis through STING-NF-κB signaling-induced macrophage pyroptosis (65). In both active RA patients and CIA mice, the levels of Pol β underwent a significant downregulation, and Pol β-deficient CIA mice exhibited exacerbated disease severity. Further investigations revealed that deficiency of Pol β promoted an augmented inflammatory response and macrophage pyroptosis in CIA mice (65). This process was mechanistically linked to enhanced DNA damage and the accumulation of dsDNA, which triggered the activation of cGAS-STING signaling. Then, NF-κB signaling was activated and NF-κB-p65 nuclear translocation was enhanced, ultimately enhancing the expression of NLRP3, IL-1β, and IL-18. These events contributed to macrophage pyroptosis and the progression of arthritis (65).

The role of the tumor suppressor gene p53 in RA pathogenesis has been explored in AIA rats (66). Overexpression of p53^R211*^ significantly alleviated arthritis symptoms and joint destruction in AIA rats, which were similar to those observed in MTX-treated rats. Beyond inhibiting T-cell activation and Th17 cell differentiation, the interaction between p53^R211*^ and TBK1 disrupted the formation of the trimeric TBK1-IRF3-STING complex. Thus, the phosphorylation and nuclear localization of IRF3 were inhibited, ultimately suppressing the autoimmunity and ameliorating inflammatory arthritis (66).

### 3.5 cGAS-STING signaling: RA onset and chronic inflammation

According to the existing literature, it seems that cGAS-STING signaling predominantly contributes to the chronic inflammation in RA. For example, there was no significant difference in the clinical scores between CIA-modeled *Sting1^+/-^* mice and wild-type mice on days 27, 30, and 33 after the first immunization, although the clinical scores of both groups were increased. Notably, the clinical scores of *Sting1^+/-^* mice were significantly lower than those of wild-type mice from day 36, indicating the promoting effects of STING during disease progression (54). Moreover, another study has revealed that joint injection of DNA fragments increases the arthritic score and hind paw volume in AIA rats, which may be due to the upregulation of cGAS-STING signaling (67). For disease onset, it has been demonstrated that TREX1 reduction and cfDNA accumulation can be risk factors for the onset of RA in elderly through activating the cGAS signaling cascade, and these characteristics have been observed in elderly RA patients and AIA rats. On day 12, the first symptom was observed in AIA rats injected intravenously with DNA fragments, indicating that intravenous injection of DNA promoted the disease onset (67). Although the pathogenesis is complex, targeting cfDNA and cGAS-STING signaling may open a new window for prevention and treatment strategies for RA.

## 4 cGAS-STING signaling: be protective in RA?

Contrary to the pathogenic effects previously mentioned, one study suggested that STING might be a “negative” regulator in the CIA model by modulating B cell functions (68). STING-deficient mice showed disease progression comparable to wild-type mice, including incidence, arthritis scores, histopathological changes, and other inflammatory parameters such as B220^+^ cells, CD4^+^ cells, and IL-6 (68). However, STING-deficient mice exhibited elevated levels of anti-CII IgG and IgG2c after three weeks of the first immunization. Gene expression profiles suggested that the disease progression in CIA mice might not have a direct correlation with IFN. Instead, the B cell receptor emerged as a significant factor, suggesting the involvement of B cells (68). B cells from STING-deficient mice exhibited enhanced survival capabilities compared to wild-type B cells, accompanied by similar cell proliferation. Furthermore, STING activation resulted in B cell death and increased Fas expression (68). Therefore, STING played a regulatory role during the development of arthritis by modulating B cell functions, and it would be interesting to explore whether the function of B cell subtypes (such as regulatory B cells) could be regulated by cGAS-STING signaling in RA. Given the conflicting results, it is reasonable to speculate that the balance of STING signaling activation in different cells may influence the disease progression, such as in B cells and FLSs. Collectively, the role of the cGAS-STING pathway in RA suggests that further investigation is needed to clarify the underlying molecular mechanisms.

## 5 cGAS-STING signaling: a target for RA treatment

**Figure 3:**
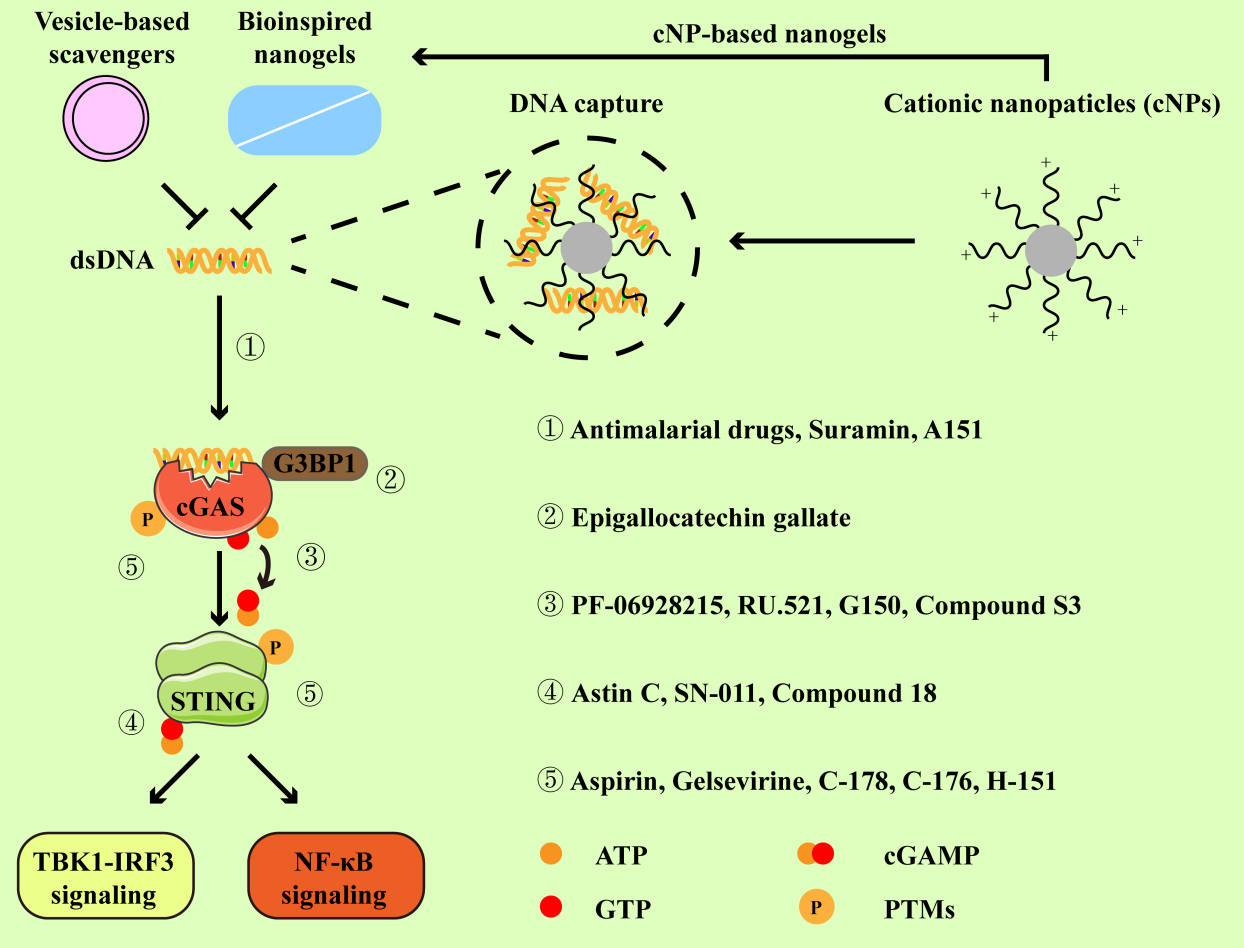
Inhibitors and their targets in the cGAS-STING pathway. Cationic nanoparticles, bioinspired nanogels, and vesicle-based scavengers serve as efficient tools to capture dsDNA, thereby preventing cGAS from activation. Representative inhibitors specifically targeting cGAS-STING pathway primarily function through the following mechanisms:① by disrupting the interaction between cGAS and dsDNA. ② by impeding the association between GRPase-activating protein-(SH3 domain)-binding protein 1 (G3BP1, a facilitator of cGAS oligomerization) and cGAS. ③ by binding to the active site of cGAS. ④ by occupying the cyclic dinucleotide (CDN)-binding site of STING. ⑤ by suppressing the activation of cGAS-STING signaling via post-translational modifications (PTMs).

According to the above description, cGAS-STING signaling primarily plays a pathogenic role in RA. Increasing studies have reported the application of pharmacologic modulators targeting cGAS-STING signaling (54, 69–71) (**Figure 3**). As the stimulator of cGAS-STING signaling, clearance of cfDNA is promising in RA therapies. Moreover, we mainly discuss representative cGAS-STING inhibitors that have been studied in RA, and other inhibitors of cGAS-STING pathway have also been summarized in **Table 1**.

**Table 1:** Other inhibitors of cGAS-STING signaling pathway and their effects.

Modulators	Targets	Effects	Cell lines/Animal models	Applications in preclinical or clinical RA	References
PF-06928215	cGAS	binds to the active site of cGAS	Sf9 cells	–	(72)
RU.521	cGAS	binds to the active site of cGAS	RAW cells and BMDMs from AGS mice	attenuates tumor-like biologic behaviors of FLSs	(40, 73)
Compound S3	cGAS	binds to the active site of cGAS	–	–	(74)
G chemotype compounds (G150)	cGAS	binds to the active site of cGAS	THP-1 cells and primary human macrophages	–	(75)
Suramin	cGAS	displaces DNA from cGAS	THP-1 cells	reduces inflammation and repairs joint destruction in CIA rats	(76, 77)
X6	cGAS	displaces DNA from cGAS	THP-1 cells and *Trex^-/-^* mice	–	(78, 79)
A151	cGAS	interacts with the dsDNA-binding domain of cGAS	THP-1 cells and *Trex^-/-^* cells	–	(80)
Cyclopeptide inhibitors (XQ2B)	cGAS	binds to the DNA binding site of cGAS	THP-1 cells and HSV-1 infected mice	–	(81)
Aspirin	cGAS	acetylates cGAS	PBMCs from AGS patients, THP-1 cells, *Trex^-/-^* mice	has been used as NSAIDs	(82, 83)
CU-32, CU-76	cGAS	–	THP-1 cells	–	(84)
Astin C	STING	binds to the CDN-binding site of STING	*Trex^-/-^* BMDMs and *Trex^-/-^* mice	–	(85)
SN-011	STING	binds to the CDN-binding pocket of STING	HSV-1-infected HFFs, 293T cells, *Trex^-/-^* BMDMs, *Trex^-/-^* mice	–	(86)
Compound 18	STING	binds to the CDN-binding site of STING	–	–	(87)
C-178, C-176, H-151	STING	binds to STING palmitoylation sites	293T cells, *Trex^-/-^* mice	inhibits the formation and activation of osteoclasts	(88, 89)
BPK-21/5	STING	binds to STING palmitoylation sites	PBMCs	–	(90)
NO_2_-FAs	STING	modifies STING by nitro-alkylation	RAW264.7 cells, THP-1 cells	–	(91)
Gelsevirine	STING	promotes K48-linked poly-ubiquitination of STING	Murine chondrocytes, OA mice	–	(92)
Nitisinone	STING	suppresses the cGAS-STING-NF-κB pathway	Murine chondrocytes, OA mice	–	(93)
TGP	STING	attenuates STING-IRF3 interaction	THP-1 cells and BMDMs	improves disease severity and reduces inflammation levels	(94, 95)

Sf9 cells, Spodoptera frugiperda cell line; BMDMs, bone marrow-derived macrophages; AGS, Aicardi-Goutières syndrome; THP-1 cells, Tohoku Hospital Pediatrics-1; T*rex* ^-/-^ mice, transcription export-deficient mice; PBMCs, peripheral blood mononuclear cells; HSV-1, herpes simplex virus-1; CDN, cyclic dinucleotide; HFFs, human foreskin fibroblasts; OA, osteoarthritis; TGP, total glucosides of paeony; -, not mentioned.

### 5.1 Scavengers of cfDNA

Cationic nanoparticles (cNPs) are composed of the diblock copolymer of poly(lactic-co-glycolic acid) (PLGA) and poly(2-(diethylamino)ethyl methacrylate) (PDMA), which have shown high DNA binding efficiency and the ability to scavenge cfDNA from RA patients (96). To decrease the risk of dissociation and toxicity, a series of silica particles grafted with PDMA (SiNP@PDMA) brush were developed. SiNP@PDMA was able to scavenge cfDNA, accompanied by the prolonged retention time in joints (97). Bioinspired nanogel composed of DNase I and a polylysine dendrimer (G3K) showed potent DNA trapping abilities while retaining nearly 90% of the biological activity of DNase I, making it effective in scavenging cfDNA (98). Recently, some novel scavengers that may be used for joint injection have been explored (99, 100). cNP-pp-PEG was designed to ensure the release of cations when polyethylene glycol (PEG) was removed by MMP2, an enzyme highly expressed in inflamed joints (99). Similarly, exosomes from M2 macrophages were modified with oligolysine and MMP-cleavable PEG, allowing the release of positively charged oligolysine to effectively scavenge cfDNA within inflamed joints (100). Although these studies have not directly explored the effects of scavengers on cGAS-STING signaling, it is possible to speculate that cGAS-STING signaling is involved in the therapeutic effects of DNA scavengers in arthritis.

### 5.2 Modulators of cGAS

The exploration of engineering and delivery mechanisms for modulators targeting cGAS, aimed at immunotherapy for RA, has been conducted in recent studies (71, 101). At first, they found that cfDNA and cGAS expression in lymph nodes or spleen from CIA mice and RA patients were upregulated. As described above, cNPs could inhibit cGAS activation and pro-inflammatory responses via scavenging cfDNA. A nanomedicine-in-hydrogel (NiH) system was devised to concurrently deliver the cGAS inhibitor (RU.521) and cNPs, which could prolong the release and retention of cNPs and RU.521 in lymphoid tissues (71, 101). Loading RU.521 to cNPs resulted in a profound reduction in *ifnb, Nos2*, and *Tnfa* in macrophages, indicating the enhanced inhibitory effects on cGAS activation and pro-inflammatory responses (101). In CIA mice, NiH ameliorated arthritis progression and reduced arthritis severity. Moreover, NiH supported the immunosuppression in CIA mice, as indicated by the reduced production of pro-inflammatory cytokines, along with a decrease in the proportions of pro-inflammatory cells and an expansion of immunomodulatory cells (71, 101). Moreover, subcutaneous administration of NiH could also achieve the above effects, not only in lymph nodes, but also in peripheral blood (71).

Anti-malarial drugs (AMDs) are commonly applied in malaria treatment and have also shown beneficial effects on autoimmune diseases over the past decades (69, 102). According to earlier research, the majority of RA patients responded to hydroxychloroquine (HCQ) treatment, as evidenced by improvements in joint score, pain, and grip strength. Only a small part of patients experienced a flare after the initial improvement (103, 104). HCQ could suppress autoimmunity by blocking MHC II-mediated autoantigen presentation and downregulating TLR signaling, and it was observed that AMDs might also regulate the activity of cGAS (102). In silico studies predicted that HCQ and other AMDs interacted with cGAS-DNA complex at a site necessary for binding to cGAS and its activation by DNA, and *in vitro* experiments confirmed that quinacrine blocked the binding between dsDNA and cGAS (78). Interestingly, the interaction between quinoline- and acridine-based antimalarial drugs (QA-AMDs) and dsDNA manifested in three modes: intercalation, groove binding, and covalent binding (105). Thus, AMDs might impair DNA-stimulated cGAS activation and subsequent pro-inflammatory cytokine expression, thereby alleviating RA progression. The combination of AMDs and other therapeutic drugs for RA therapy has been explored (106, 107). Compared with the methotrexate/leflunomide (MTX/LEF) group, MTX/HCQ-treated patients had a higher level of remission rate, accompanied by a rapid remission. Remarkably, more patients treated with MTX/HCQ were able to withdraw glucocorticoid exposure than those treated with MTX/LEF (107). A novel Pluronic^®^ F-127 nanomicelle co-loaded with HCQ and MTX exhibited therapeutic effects against murine arthritis, which efficiently suppressed the osteoclastogenesis (106). Now, there are more potent anti-inflammatory drugs and biological agents, which may limit the application of AMDs in RA treatments (3).

### 5.3 Modulators of STING

Recent studies have delved into compounds that modulate STING activity and their potential utilization in the treatment of RA (54, 70). C-176, a covalent small-molecule inhibitor with the ability to antagonize STING and suppress IFN-I production, has been demonstrated to attenuate the disease development and reduce the bone erosion in CIA mice. Mice treated with of C-176 displayed reduced disease scores, accompanied by the decreased level of tartrate-resistant acid phosphatase-expressing osteoclasts (88). There was a study that confirmed the efficacy of polyethyleimine-polydopamine (PEI-PDA)@C-176 NPs in adsorbing DNA and inhibiting STING, suggesting their potential application in the treatment of RA (54). *In vitro*, PEI-PDA@C-176 NPs suppressed the phosphorylation and activation of TBK1 and IRF3, leading to a notable reduction in IFN-β, TNF-α, and IL-6 expression in human primary FLSs (54). In dsDNA-induced arthritis and CIA models, PEI-PDA@C-176 NPs effectively alleviated inflammation, which was evidenced by improved ankle swelling, reduced histological scores, and other disease indexes (54). Mechanistically, these therapeutic effects were dependent on the STING signaling pathway, because PEI-PDA@C-176 NPs only slightly reduced the clinical score in CIA-modeled *Sting1* ^+/−^ mice without improvements in synovitis (54).

Triptolide (TP), the pharmacological component of the herb Tripterygium wilfordii Hook F (TWHF), has shown therapeutic effects in RA. Previous studies revealed that TP exerted therapeutic effects in RA by targeting RA-associated proteins (such as NF-κB, MMP-9 and JNK), accompanied by reduced activity of FLSs and Th17 differentiation (108–110). Interestingly, a recent study has reported that TP can exerts immunomodulatory effects by regulating cGAS-STING signaling (70). FDL@TP is formed by encapsulating TP with an amphiphilic polymer (FDL) composed of folic acid and lauric acid, which has been investigated in RA treatment (70). FDL@TP exhibited the ability to specifically target joints and efficiently promoted the uptake of TP by M1 macrophages. FDL@TP reduced the expression of cGAS and STING, which further led to the reduction in TNF-α, IL-1β, and IL-6 production (70). Importantly, FDL@TP was more effective than the same dose of TP alone in controlling inflammatory responses, also with reduced side effects (70). In recent years, nanodrugs based on TP have been developed for RA therapy, including folate-modified TP liposomes, TP nanoemulsion gel, and TP-carrying dendritic cell-derived exosomes, which are promising for localized treatment with reduced toxicity (111–113). However, their effects on cGAS-STING signaling remain to be investigated.

### 5.4 Other modulators of cGAS-STING signaling pathway

Additional modulators primarily target the downstream signaling of cGAS-STING signaling pathway, and some of them have been studied in animal models. CS12192, a small molecule inhibitor of JAKs, primarily targets JAK3/1 and has inhibitory effects on TBK1. This leads to the reduced activation of IRF3 and downregulation of IFN-I. In preclinical models of arthritis, CS12192 has been reported to ameliorate the disease severity and bone destruction, along with immunomodulatory effects such as the suppression of CD4^+^ T cell activation and reduction in pro-inflammatory cytokine production (114). Itaconate (ITA) is an endogenous metabolite from the tricarboxylic acid cycle, which has been confirmed to suppress osteoarthritis by reducing the activation of STING-dependent NF-κB pathway. In a preclinical model of RA, ITA reduced arthritis severity and bone erosion by suppressing the proliferation and migration of FLSs. In addition, mice lacking immunoresponsive gene 1 (Irg1) failed to express endogenous ITA and showed more severe arthritis, underscoring the role of ITA in modulating inflammation (115, 116). Auranofin, a gold compound approved by U.S. Food and Drug Administration for RA treatment, has been demonstrated to act as a small-molecule inhibitor of IRF3 (117). In this study, they demonstrated that auranofin promoted the degradation of IRF3 by inducing cellular autophagy, thereby suppressing the transcriptional activities of IRF3 (117).

Although these modulators exhibit potential in inhibiting RA progression, efficacy improvement remains an important task. Moreover, considering the long course of RA, strategies to prolong the release and consumption of these inhibitors (such as the use of a hydrogel system) are important areas for future clinical research.

## 6 Conclusions and prospects

Hitherto, it is clear that innate immunity holds a pivotal position in the development of autoimmune diseases. In the past few years, there has been a significant increase in interest and understanding surrounding the cGAS-STING signaling, which is a main danger-sensing mechanism of innate immunity. Although there is still much to be learned in autoimmune diseases, it is promising to target cGAS-STING signaling for treatments given the abnormal activation of cGAS-STING signaling by cfDNA (16).

With the deepening of research, the significance of cGAS-STING signaling in rheumatoid arthritis has been gradually recognized. For instance, TNF has been shown to induce the release of mitochondrial DNA (mtDNA), which then activates cGAS-STING-mediated IFN responses, contributing to the progression of arthritis. This effect can be suppressed by FTO knockdown through a CMPK2-dependent manner (57, 61). In addition, dsDNA-induced cGAS-STING signaling has been shown to promote the development of arthritis through the induction of inflammatory factors in FLSs, accompanied by enhanced migration and invasion (63, 65). Notably, there was a study pointed out that deficiency of STING promoted CIA progression by enhancing B cell survival and autoantibody production (68). However, fewer studies are available to further confirm the roles of cGAS-STING signaling in the differentiation and function of B cells during RA development.

Considering the critical roles of TNF in DNaseII reduction, mtDNA release, and cGAS expression, TNF inhibitors may also inhibit the cGAS-STING signaling. For cfDNA clearance, several scavengers (such as cNPs and cNP-pp-PEG) have been developed and exhibit strong DNA-capturing abilities (96, 99). Current pharmacological modulators targeting cGAS-STING signaling mainly include NiH, PEI-PDA@C-176 NPs, and FDL@TP, and these inhibitors have been confirmed to be effective in both *in vitro* experiments and murine arthritis (54, 70, 71). Due to challenges in medicinal chemistry, one antagonist (VENT-03, targeting cGAS) has been advanced into phase I clinical trials, which aims to evaluate the safety of VENT-03 in healthy volunteers, and subsequent trial plans will target the treatment of autoimmune diseases (118). Thus, there is still a lack of evidence in treating human RA. Collectively, future explorations should pay more attention to the following fields: 1) molecular mechanisms regulating the activation of cGAS-STING signaling during RA; 2) the development of novel drugs targeting the cGAS-STING signaling pathway, with emphasis on clinical applications. These efforts may provide new insights into the therapies for RA and other autoimmune diseases.
